# Supplementary material for: Tuning Fairness by Balancing Target Labels
Source: Front Artif Intell. 2020 May 12;3:33. doi: 10.3389/frai.2020.00033 (PMC7861271; doi:10.3389/frai.2020.00033)
Supplement: Supplementary file 1 [file Data_Sheet_1.PDF]

Supplementary Material

1 PROOF OF THEOREM 1

Let $\eta(x,s) = P(y=1|x,s)$ be the distribution of the training data. Let $\bar{\eta}(x,s) = m_s \cdot \eta(x,s) + b_s$, where

$$m_s = P(\bar{y}=1|y=1,s) - P(\bar{y}=1|y=0,s)$$
$$= 1 - P(\bar{y}=0|y=1,s) - P(\bar{y}=1|y=0,s) \quad \text{(S1)}$$
$$b_s = P(\bar{y}=1|y=0,s) \quad \text{(S2)}$$

So, $\bar{\eta}(x,s) = P(\bar{y}=1|x,s)$. Let y denote the *hard* labels for η: $y = \mathbb{I}\left[\eta > \frac{1}{2}\right]$ and $\bar{y}$ be the hard labels for $\bar{\eta}$: $\bar{y} = \mathbb{I}\left[\bar{\eta} > \frac{1}{2}\right]$.

THEOREM 1. *The probability that y and $\bar{y}$ disagree ($y \neq \bar{y}$) for any input x in the dataset is given by:*

$$\mathbb{P}(y \neq \bar{y}|s) = \mathbb{P}\left(\left|\eta(x,s) - \tfrac{1}{2}\right| < t_s\right) \quad \text{(S3)}$$

where

$$t_s = \left|\frac{m_s + 2b_s - 1}{2m_s}\right| . \quad \text{(S4)}$$

PROOF. The decision boundary that lets us recover the true labels is at $\frac{1}{2}$ (independent of s). So, for the shifted distribution, $\bar{\eta}$, this threshold to get the true labels would be at $\frac{1}{2} \cdot m_s + b_s$ (it depends on s now). If we however use the decision boundary of $\frac{1}{2}$ for $\bar{\eta}$, to make our predictions, $\bar{y}$, then this prediction will sometimes not correspond to the true label, $y \neq \bar{y}$. When does this happen?

Let d_s be the new decision boundary: $d_s = \frac{1}{2} \cdot m_s + b_s$. There are two possibilities to consider here: either $\frac{1}{2} < d_s$ or $\frac{1}{2} > d_s$ (for $d_s = \frac{1}{2}$, the decision boundaries are the same and nothing has to be shown). The problem, $y \neq \bar{y}$, appears then exactly when the value of $\bar{\eta}$ is between the two boundaries:

$$\text{if } d_s > \tfrac{1}{2}: \quad d_s > \bar{\eta}(x,s) > \tfrac{1}{2} \quad \text{(S5)}$$
$$\text{if } d_s < \tfrac{1}{2}: \quad d_s < \bar{\eta}(x,s) < \tfrac{1}{2} \quad \text{(S6)}$$

Expressing this in terms of η and simplifying leads to (if m_s is negative, then the two cases are swapped, but we still get both inequalities):

$$\text{if } d_s > \tfrac{1}{2}: \quad \frac{1}{2} > \eta(x,s) > \frac{1-2b_s}{2m_s} \quad \text{(S7)}$$
$$\text{if } d_s < \tfrac{1}{2}: \quad \frac{1}{2} < \eta(x,s) < \frac{1-2b_s}{2m_s} \quad \text{(S8)}$$

This can be summarized as

$$\left|\eta(x,s) - \frac{1}{2}\right| < \left|\frac{1}{2} - \frac{1-2b_s}{2m_s}\right| . \quad \text{(S9)}$$

Let t_s denote the term on the right side of this inequality (i.e. the "threshold" that determines whether $y = \bar{y}$ or not). Then

$$t_s = \left| \frac{1}{2} - \frac{1-2b_s}{2m_s} \right| = \left| \frac{m_s + 2b_s - 1}{2m_s} \right| \; . \tag{S10}$$

So, we have: $\left|\eta(x,s) - \frac{1}{2}\right| < t_s = \left|\frac{m_s+2b_s-1}{2m_s}\right|$. This leads directly to the statement we wanted to prove:

$$P(y \neq \bar{y}|s) = P\left(\left| \eta(x,s) - \frac{1}{2} \right| < t_s \right) \; . \tag{S11}$$

2 FINDING MINIMAL T_S

We express t_s in terms of PR_b^s and PR_t.

$$t_s = \begin{cases} \frac{1}{2}\frac{PR_b^s - PR_t}{PR_t} & \text{if } PR_t > PR_b^j \\ \frac{1}{2}\frac{PR_t - PR_b^s}{1-PR_t} & \text{otherwise.} \end{cases} \tag{S12}$$

Without loss of generality, we assume $PR_b^0 < PR_b^1$. As mentioned in the main text, PR_t should be between PR_b^0 and PR_b^1 to minimize both t_s. If that is the case, then we get

$$t_{s=0} = \frac{1}{2}\frac{PR_t - PR_b^0}{1 - PR_t} \tag{S13}$$

$$t_{s=1} = \frac{1}{2}\frac{PR_b^1 - PR_t}{PR_t} \; . \tag{S14}$$

If we further assume $PR_b^1 < \frac{1}{2}$, then we also have $PR_t < \frac{1}{2}$ and thus $PR_t < 1 - PR_t$. This implies that the denominator of $t_{s=1}$ is smaller and that, in turn, $t_{s=1}$ grows faster. This faster growth means that when minimizing $t_{s=0} + t_{s=1}$, we have to concentrate on $t_{s=1}$. The minimum is then such that $t_{s=1}$ is 0, i.e. $PR_t = PR_b^1$.

3 PROOF OF THEOREM 2

We are given a dataset $\mathcal{D} = \{(x_i, y_i)\}_i$, where the x_i are vectors of features and the y_i the corresponding labels. We refer to the tuples (x, y) as the *samples* of the dataset. The number of samples is $N = |\mathcal{D}|$.

We assume binary labels ($y \in \{0, 1\}$) and thus can form the (disjoint) subsets $\mathcal{Y}^0$ and $\mathcal{Y}^1$ with

$$\mathcal{Y}^j = \{(x,y) \in \mathcal{D} | y = j\} \quad \text{with } j \in \{0,1\} \; . \tag{S15}$$

Furthermore, we associate each sample with a classification $\hat{y} \in \{0, 1\}$. The task of making the classification $\hat{y} = 0$ or $\hat{y} = 1$ can be understood as putting each sample from $\mathcal{D}$ into one of two sets: $\mathcal{C}^0$ and $\mathcal{C}^1$, such that $\mathcal{C}^0 \cup \mathcal{C}^1 = \mathcal{D}$ and $\mathcal{C}^0 \cap \mathcal{C}^1 = \emptyset$.

We refer to the set $\mathcal{A} = (\mathcal{C}^0 \cap \mathcal{Y}^0) \cup (\mathcal{C}^1 \cap \mathcal{Y}^1)$ as the set of correct (or **a**ccurate) predictions. The *accuracy* is given by $acc = N^{-1} \cdot |\mathcal{A}|$. From the definition it is clear that $0 \leq acc \leq 1$.

Definition 1.

$$r_a := \frac{|\mathcal{Y}^1|}{|\mathcal{D}|} = \frac{|\mathcal{Y}^1|}{N} \tag{S16}$$

is called the *acceptance rate* of the dataset $\mathcal{D}$.

Definition 2.

$$\hat{r}_a = \frac{|\mathcal{C}^1|}{|\mathcal{D}|} = \frac{|\mathcal{C}^1|}{N} \tag{S17}$$

is called the *target rate* of the predictions.

THEOREM 2. *For a dataset with the acceptance rate r_a and corresponding predictions with a target rate of $\hat{r}_a$, the accuracy is limited by*

$$acc \leq 1 - |\hat{r}_a - r_a| \ . \tag{S18}$$

PROOF. We first note that by multiplying by N, the inequality becomes

$$|\mathcal{A}| \leq N - \big||\mathcal{C}^1| - |\mathcal{Y}^1|\big| \ . \tag{S19}$$

We will choose the predictions $\hat{y}$ that achieve the highest possible accuracy (largest possible $\mathcal{A}$) and show that this can never exceed $1 - |\hat{r}_a - r_a|$. As the set $\mathcal{Y}^1$ contains all samples that correspond to $y = 1$, we try to take as many samples from $\mathcal{Y}^1$ for $\mathcal{C}^1$ as possible. Likewise, we take as many indices as possible from $\mathcal{Y}^0$ for $\mathcal{C}^0$.

We consider three cases: $\hat{r}_a = r_a$, $\hat{r}_a < r_a$ and $\hat{r}_a > r_a$. The first case is trivial; we have $\left|\mathcal{C}^1\right| = \left|\mathcal{Y}^1\right|$ and thus are able to set $\mathcal{C}^1 = \mathcal{Y}^1$, $\mathcal{C}^0 = \mathcal{Y}^0$ and achieve perfect accuracy ($acc \leq 1$).

For $\hat{r}_a < r_a$, we have $\left|\mathcal{C}^1\right| < \left|\mathcal{Y}^1\right|$ and thus have more samples available with $y = 1$ than we would optimally need to select for $\mathcal{C}^1$. There are two terms to consider that make up the definition of $\mathcal{A}$: $\mathcal{C}^0 \cap \mathcal{Y}^0$ and $\mathcal{C}^1 \cap \mathcal{Y}^1$. The intersection of these two terms is empty because $\mathcal{C}^0 \cap \mathcal{C}^1 = \emptyset$. Thus,

$$|\mathcal{A}| = \left|(\mathcal{C}^0 \cap \mathcal{Y}^0) \cup (\mathcal{C}^1 \cap \mathcal{Y}^1)\right| = \left|(\mathcal{C}^0 \cap \mathcal{Y}^0)\right| + \left|(\mathcal{C}^1 \cap \mathcal{Y}^1)\right| \ . \tag{S20}$$

Selecting samples from $\mathcal{Y}^1$ for $\mathcal{C}^0$ will only *decrease* the first term, so for maximum accuracy, it is fine to take as many samples from $\mathcal{Y}^1$ for $\mathcal{C}^1$. Taking all available samples from $\mathcal{Y}^1$ such that $\mathcal{C}^1 \supset \mathcal{Y}^1$, there is still space left in $\mathcal{C}^1$ which we will have to fill with samples with $y = 0$. Thus, we have $\mathcal{C}^1 \cap \mathcal{Y}^1 = \mathcal{Y}^1$. For $\mathcal{C}^0$, we have enough $y = 0$ such that $\mathcal{C}^0 \subset \mathcal{Y}^0$ and $\mathcal{C}^0 \cap \mathcal{Y}^0 = \mathcal{C}^0$. This is the largest we can make these intersections. Putting everything together:

$$\begin{aligned} \left|\mathcal{A}^{optimal}\right| &= \left|(\mathcal{C}^0 \cap \mathcal{Y}^0)\right| + \left|(\mathcal{C}^1 \cap \mathcal{Y}^1)\right| = \left|\mathcal{C}^0\right| + \left|\mathcal{Y}^1\right| \\ &= N - \left|\mathcal{C}^1\right| + \left|\mathcal{Y}^1\right| = N - \left(\left|\mathcal{C}^1\right| - \left|\mathcal{Y}^1\right|\right) \ . \end{aligned} \tag{S21}$$

For $\hat{r}_a > r_a$, the roles of $\mathcal{C}^0$ and $\mathcal{C}^1$ are reversed and thus, the signs in the equation are inverted:

$$\left|\mathcal{A}^{optimal}\right| = N - \left(\left|\mathcal{Y}^1\right| - \left|\mathcal{C}^1\right|\right) . \tag{S22}$$

This proves the claim.

COROLLARY 2.1. *Given a dataset that consists of two subsets $\mathcal{S}_0$ and $\mathcal{S}_1$ ($\mathcal{D} = \mathcal{S}_0 \cup \mathcal{S}_1$) where p is the ratio of $|\mathcal{S}_0|$ to $|\mathcal{D}|$ and given corresponding acceptance rates r_a^0 and r_a^1 and predictions with target rates $\hat{r}_a^0$ and $\hat{r}_a^1$, the accuracy is limited by*

$$acc \leq 1 - p \cdot \left|\hat{r}_a^0 - r_a^0\right| - (1-p) \cdot \left|\hat{r}_a^1 - r_a^1\right| \ . \tag{S23}$$

Example 1. We consider the case where S_0 (which could for example be all data points for female individuals) makes up 30% of the dataset; so $p = 0.3$. Further, we say that for S_0 we have an acceptance rate of 10% ($r_a^0 = 0.1$) and for S_1, 50% ($r_a^1 = 0.5$). If we then set both target rates to the same value t ($\hat{r}_a^0 = \hat{r}_a^1 = t$), with $t = 0.3$, then the highest accuracy that can be achieved is 0.8 or 80%.

Fig S1 shows the achievable accuracy for different values of t in blue: We can see that we can achieve the highest accuracy for $t = r_a^1 = 0.5$, namely 88%. The plot in orange shows the achievable accuracy for $p = 0.5$, i.e., when the two subsets have the same size. In this case, all target rates between r_a^0 and r_a^1 give equal results, namely 80%.

4 ILLUSTRATION OF RESTRICTIONS ON PR

We start by setting a target rate r_t:

$$P(\bar{y} = 1 | s = 0) \stackrel{!}{=} r_t \quad \text{and} \quad P(\bar{y} = 1 | s = 1) \stackrel{!}{=} r_t \tag{S24}$$

This leads us to the following constraint for $s' \in \{0, 1\}$:

$$\begin{aligned} r_t &= P(\bar{y} = 1 | s = s') \\ &= \sum_y P(\bar{y} = 1 | y, s = s') P(y | s = s') \end{aligned} \tag{S25}$$

For $P(y|s = s')$ we will put in the value at which we want our constraint to hold. We denote $P(y = 1|s = j)$ as the base rate r_b^j which we estimate from the training set. Plugging this in, we are left with

$$\begin{aligned} r_t = \ & P(\bar{y} = 1 | y = 0, s = 0) \cdot (1 - r_b^0) \\ & + P(\bar{y} = 1 | y = 1, s = 0) \cdot r_b^0 \end{aligned} \tag{S26}$$

$$\begin{aligned} r_t = \ & P(\bar{y} = 1 | y = 0, s = 1) \cdot (1 - r_b^1) \\ & + P(\bar{y} = 1 | y = 1, s = 1) \cdot r_b^1 \ . \end{aligned} \tag{S27}$$

This is a system of linear equations with two equations and four free variables. There is thus still considerable freedom in how we want our constraint to be realized. The freedom that we have here concerns how strongly the accuracy will be affected.

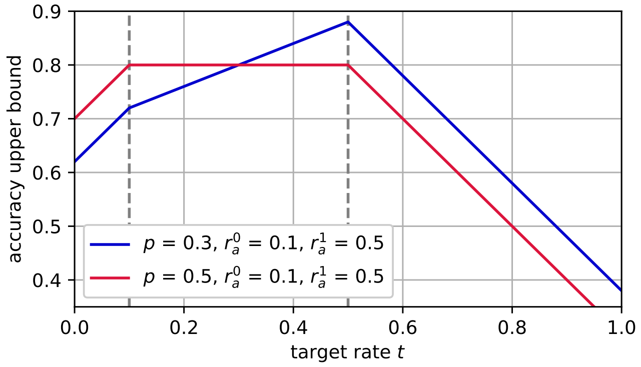

Figure S1. Example

If we set $P(\bar{y} = 1|y = 1, s)$ to 0.5, then we express the fact that a train label of 1 only implies a target label of 1 in 50% of the cases. In order to minimize the effect on accuracy, we make $P(\bar{y} = 1|y = 1, s)$ as high as possible and $P(\bar{y} = 1|y = 0, s)$ as low as possible.

We solve for $P(\bar{y} = 1|y = 0, s = j)$:

$$P(\bar{y} = 1|y = 0, s = j) = \frac{r_b^j}{1 - r_b^j}\left(\frac{r_t}{r_b^j} - P(\bar{y} = 1|y = 1, s = j)\right) . \tag{S28}$$

However, we can set $P(\bar{y} = 1|y = 0, s = j)$ to 0 only if that does not imply $P(\bar{y} = 1|y = 1, s = j)$ will be greater than 1. This would happen if r_t/r_b^j were greater than 1.

Figure S2 illustrates this. In the upper part of the figure we have r_t/r_b^j less than 1. In this case

5 SUPPLEMENTARY TABLES AND FIGURES

5.1 Figures

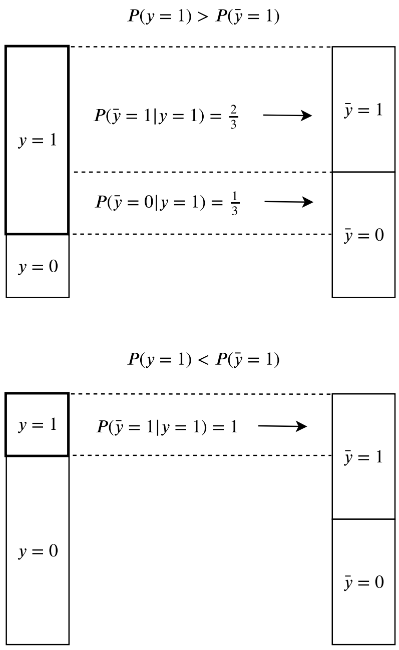

Figure S2. Illustration of demographic parity with target labels. In the situation in the upper part, $P(\bar{y} = 1|y = 1)$ cannot be set to 1, because there are more samples with $y = 1$ than there are $\bar{y} = 1$. In the situation in the lower part, $P(\bar{y} = 1|y = 1)$ can be set to 1.
